# Exosomal GAPDH from Proximal Tubule Cells Regulate ENaC Activity

**DOI:** 10.1371/journal.pone.0165763

**Published:** 2016-11-01

**Authors:** Kishore Kumar Jella, Ling Yu, Qiang Yue, Daniel Friedman, Billie J. Duke, Abdel A. Alli

**Affiliations:** 1 Department of Physiology and Functional Genomics and Department of Medicine Division of Nephrology, Hypertension, and Renal Transplantation, University of Florida College of Medicine, Gainesville, Florida, United States of America; 2 Department of Radiation Oncology, Emory University School of Medicine, Atlanta, Georgia; 3 Department of Physiology, Emory University School of Medicine, Atlanta, Georgia; 4 College of Resources and Environmental Sciences, Nanjing Agricultural University, Nanjing, Jiangsu Province, China; Texas Technical University Health Sciences Center, UNITED STATES

## Abstract

Exosomes are nanometer-scale, cell-derived vesicles that contain various molecules including nucleic acids, proteins, and lipids. These vesicles can release their cargo into adjacent or distant cells and mediate intercellular communication and cellular function. Here we examined the regulation of epithelial sodium channels in mpkCCD cells and distal tubule Xenopus 2F3 cells by exosomes isolated from proximal tubule LLC-PK1 cells. Cultured mpkCCD cells were stained with CTX coupled to a green fluorophore in order to label the cell membranes and freshly isolated exosomes from LLC-PK1 cells were labeled with the red lipophilic dye PKH26 in order to visualize uptake of exosomes into the cells. Single-channel patch clamp recordings showed the open probability of ENaC in Xenopus 2F3 cells and in freshly isolated split-open tubules decreased in response to exogenous application of exosomes derived from LLC-PK1 proximal tubule cells. Active GAPDH was identified within exosomes derived from proximal tubule LLC-PK1 cells. The effect on ENaC activity in Xenopus 2F3 cells was blunted after application of exosomes transfected with the GAPDH inhibitor heptelidic acid. Also, we show GAPDH and ENaC subunits associate in mpkCCD cells. These studies examine a potential role for exosomes in the regulation of ENaC activity and examine a possible mechanism for communication from proximal tubule cells to distal tubule and collecting duct cells.

## Introduction

Each segment of the nephron expresses epithelial cells with unique characteristics and specialized functions. Epithelial cells lining the proximal tubule are responsible for reabsorption of approximately two-thirds of the glomerular filtrate. The fine-tuning for sodium reabsorption occurs in the distal tubule and collecting duct. Communication from the proximal tubule to the distal tubule can occur by paracrine agents. For example, proximally-derived, luminal adenosine-5′-triphosphate (ATP) may act as a signaling molecule in the distal nephron to regulate transport of electrolytes and maintain cell volume [1].

Another mechanism for communication from the proximal tubule to the distal tubule may involve exosomes [2]. Exosomes are specialized nanometer-scale membranous vesicles derived from cells and are present in biological fluids including blood and urine. Exosomes are more than vesicles of exported waste products since they can allow mRNAs, microRNAs, DNA, and proteins to be transferred between cells [3, 4]. Although exosomes have been shown to play a role in health and disease the molecular mechanisms involved remain largely unknown. Van Balkom et al. reviewed the potential impact of exosome research in the fields of nephrology and renal physiology [5].

The epithelial sodium channel (ENaC) plays an important role in the transport of sodium across the luminal membrane of distal tubule and collecting duct cells. The physiological significance of ENaC in the kidney is underscored by its role in maintaining total body sodium homeostasis and blood pressure control. ENaC usually exhibits long mean open and closed times, low single-channel conductance, and sensitivity to the diuretic amiloride at nanomolar concentrations [6].

ENaC insertion into the membrane and the number of functional channels expressed is under the control of hormones such as aldosterone and vasopressin. The rate of ENaC turnover is affected by Nedd4-2 mediated ubiquination leading to lysosomal and/or proteasomal degradation pathways [7]. The open probability (i.e. gating) of ENaC is affected by different determinants including proteolytic activity [8–15], phospholipase C [16, 17], changes in oxidative stress [18], changes in intracellular calcium concentration [19], and anionic phospholipid phosphates [16, 20, 21].

We and others have demonstrated a role for the actin cytoskeleton in regulating ENaC activity in the kidney [19, 22–27]. ENaC interacts with several actin cytoskeleton associated proteins including MARCKS, filamin, and fodrin [19, 23, 28]. The actin cytoskeleton serves as an organizing center to keep ENaC and key regulators including other proteins and lipids in close proximity. In particular, the glycolytic enzyme glyceraldehyde-3-phosphate dehydrogenase (GAPDH) directly binds cytoskeletal elements. Minaschek et al showed the soluble fraction of GAPDH is homogeneously distributed across the cytoplasm while the insoluble form of GAPDH associates with the actin cytoskeleton [29]. Cueille et al proposed a role for MAP1B in keeping GAPDH close to the cytoskeleton to allow energy provision for microtuble assembly and microfilament formation [30].

Patterson et al showed GAPDH contributes to local NADH+ and regulates IP3R-mediated Ca2+ signaling [31]. Multiple studies have shown ENaC is regulated by calcium [19, 32, 33]. We previously showed calcium in concert with calmodulin inhibits the MARCKS mediated PIP2 dependent regulation of ENaC [19]. We also showed the calcium dependent activation of CaMKII plays a role in the reorganization of the cytoskeleton and decrease in ENaC activity [19].

The present study examines the ability of exosomes from proximal tubule cells to regulate ENaC activity in the distal tubule and collecting duct. We present a possible mechanism for the regulation of ENaC activity that involves the exosomal delivery of GAPDH.

## Methods

### Cell culture

Mouse mpkCCD cells, a cortical collecting duct principal cell line were originally obtained from Dr. Alain Vandewalle (Institut National de la Santé et de la Recherche Médicale Unité; France). mpkC[^#CD cells were cultured in DMEM and Ham's F-12 medium (1:1 mixture) (GIBCO; Grand Island, NY) supplemented with 20 mM HEPES, 2 mM l-glutamine, 1 nM triiodothyronine, 50 nM dexamethasone, 0.1% penicillin-streptomycin, and 2% heat-inactivated FBS. Media was replaced 3 times/week and cells were maintained at 5% CO_2_ and 37°C. Experiments were conducted using cells between passages 28 and 37.

Xenopus 2F3 cells, a distal nephron cell line were provided as a generous gift from Dr. Dale Benos (University of Alabama). 2F3 cells were cultured in DMEM and Ham's F-12 medium (1:1 mixture) (GIBCO), supplemented with 1.5 μM aldosterone (ACROS), 1.0% streptomycin and 0.6% penicillin (GIBCO), and 5% FBS (GIBCO) at pH 7.4. Media was replaced 3 times/week and cells were maintained at 4% CO_2_ and 26°C. Only cells between passages 99 and 106 were used for experiments.

LLC-PK1 cells, a proximal tubule cell line were obtained from the American Type Culture Collection (Manassas, VA). LLC-PK1 cells were cultured in DMEM medium (GIBCO) supplemented with 0.5% penicillin/streptomycin (GIBCO), 2 mM L-glutamine, and 10% FBS. Media was replaced 3 times/week and cells were maintained at 5% CO_2_ and 37°C.

Cells were maintained in plastic tissue culture flasks before being plated and grown to confluency on permeable transwell inserts (12 and 24 mm) for protein biochemistry experiments or glutaraldehyde-fixed, collagen-coated polyester (Millipore; Danvers, MA) attached to the bottom of Lucite rings for patch-clamp experiments.

### Animal Studies

SV129 wild-type mice (The Jackson Laboratory, Bar Harbor, Maine) of either sex between the age of 8 and 12 weeks were maintained on a 12 hour light/12 hour dark cycle and fed a standard laboratory chow and tap water *ad libitum* before euthanasia. Mice were euthanized by an overdose of ketamine/xylazine and after reaching a surgical plane of anesthesia (assessed by toe pinch), cervical dislocation. All animal studies were approved by the Emory Institutional Care and Use Committee.

### Isolation of exosomes

Exosomes were isolated as described by Jella et al [34] with the following modifications. Conditioned media from cell monolayers was collected every 48 hours from the inside (apical) or outside (basolateral) compartments while the cells were grown on permeable transwell inserts. The conditioned media was centrifuged for 10 minutes at 1000 X g to remove dead cells and debris. In order to remove particles bigger than 220 nm, the supernatants were filtered through 0.22 μm Nalgene filters. The supernatant was further subjected to centrifugation at 10000 X g for 30 minutes to remove any debris left over after filtration. The supernatant was subject to ultracentrifugation at 118000 X g for 70 minutes at 4°C using a fixed-angle rotor Ti-70 (Beckman Coulter, Inc., CA). In the final step, the pellets were washed with PBS and subjected to ultracentrifugation again at 118000 X g for 70 minutes at 4°C using a fixed-angle rotor Ti-70. The pelleted exosomes were lysed in RIPA buffer (Pierce; Rockford IL) for protein biochemistry studies or resuspended in PBS for uptake assays and functional studies. Exosomes were stored at -80°C in PBS supplemented with DMSO (0.5% final concentration) in order to prevent lysis from the formation of ice crystals.

### Nanosight

Both size distribution and the concentration of exosomes reconstituted in PBS were analyzed using a Nanosight NS300 system (Malvern Instruments, UK). One microliter of the exosome suspension obtained from conditioned media from the apical or basolateral compartments of proximal tubule cells cultured on permeable transwell inserts was mixed with 1 ml of PBS and introduced into the Nanosight system. The exosomes that passed through a chamber with a laser beam were visualized through a 20X objective lens connected with a video camera. The instrument was equipped with Nanoparticle Tracking Analysis software, which specifically tracks the Brownian motion of the vesicles in solution.

### Electron microscopy

Exosomes suspended in PBS were placed on 400-mesh carbon-coated copper grids and incubated for 5 minutes for the particles to adhere to the carbon film. For negative staining, 5 μl of 1% aqueous phosphotungstic acid (pH 6.5) was applied onto the grid immediately after water removal and then removed with filter paper after 30 seconds. The grid was allowed to dry completely before viewing on a JEOL JEM-1400 transmission electron microscope (JEOL Ltd.) equipped with a Gatan US1000, 2k x 2k CCD camera (Gatan Inc.).

### Exosome labeling for uptake assay

Freshly isolated exosomes were labeled with the PKH26 red fluorescent cell marker kit (Sigma Aldrich; St. Louis, MO) according to the manufactures instructions and the following modifications. In the final isolation step the exosomes were resuspended in PBS and further mixed with diluent C and the red fluorescent lipophilic dye, PKH26, supplied by the manufacturer. The excess dye was removed by adding 1% BSA solution. The samples were transferred to 30,000 MW vivaspin filters (Sartorius Stedim North America) and washed three times. The samples were transferred to new vivaspin filters and washed with 5 ml of cell culture media. The final exosome pellet was reconstituted in 2 ml of serum free media before adding it to cells. Exosomes were incubated on the cells for one hour and after one hour the exosomes were removed from the cells by washing the cells three times with PBS. The cells were then incubated with 4% paraformaldehyde solution for 15 min and washed three more times. The membrane was then mounted on a glass slide with mounting medium containing DAPI. The slides were then subject to analysis by confocal microscopy. Microscopy images were obtained using a Leica SP8 multiphoton microscope using an HC PL APO 63X 1.40 NA oil objective.

### GAPDH activity assay

A GAPDH activity assay kit (BioVision Milpitas, CA) was used to measure GADPH activity in exosomes. Briefly, the final volume of exosomes lysed in RIPA buffer was adjusted to 50 μL by adding ice cold GAPDH assay buffer. The samples were incubated on ice for 10 minutes. Each exosome sample, positive control, and standard was mixed and incubated with 50 μl of reaction mixture composed of GAPDH assay buffer, developer, and substrate for an additional 5 minutes before being added to a 96 well plate. The plate was measured using a Tecan Safire 96 well plate reader (Switzerland) at 450 nm in kinetic mode for 40 minutes at 37°C. An NADH standard curve was generated and the background control optical density value was subtracted from all sample recordings. The ΔOD was calculated for each test sample and positive control absorbance readings from the first and last time points. The amount of NADH generated by GAPDH activity during the reaction time was calculated by applying the ΔOD value to the NADH standard curve. Sample GAPDH activity in Units/ml was calculated by dividing the amount of NAHD generated during the reaction time by the product of ΔT and total sample volume. For GAPDH inhibition studies, Heptelidic acid (Koningic acid) (Abcam; Cambridge, MA) was transfected into exosomes using the Exo-Fect exosome reagent (System Biosciences, LLC; Palo Alto, CA).

### Transepithelial current measurements

An epithelial voltmeter (EVOM, World Precision Instruments; Sarasota, FL) was used to measure transepithelial voltages and resistances across confluent cell monolayers after application of exosomes to the apical side of permeable transwell inserts under sterile conditions. Ohm’s law was used to calculate transepithelial current while correcting for the surface area of the permeable transwell inserts used to culture the cells and was expressed as microamperes per square centimeter. Amiloride (0.5 μM) was applied to the apical side of the cell monolayers at the end of each experiment in order to determine amiloride-sensitive current.

### Single-channel patch clamp

Micropipettes had a resistance of 6–10 MΩ after being pulled from filamented borosilicate glass capillaries (TW-150F, World Precision Instruments) using a two-stage vertical puller (Narishige, Tokyo, Japan). Xenopus 2F3 cells were cultured on glutaraldehyde-fixed, collagen-coated polyester filters (Millipore) attached to the bottom of Lucite rings. At room temperature, Xenopus 2F3 cells were visualized with Hoffman modulation optics and the micropipette tip was positioned on top of the cell before making contact with the cell surface. Using a syringe negative pressure was applied to obtain a seal with a resistance of 10–20 GΩ. Physiological amphibian saline titrated with 0.1 N NaOH or HCl to a pH of 7.3–7.4 consisted of (in mM) 3.4 KCl, 95 NaCl, 0.8 MgCl_2_, 0.8 CaCl_2_, and 10 HEPES and was used for the extracellular bath and patch pipette solutions. The cell-attached patch configuration was used for all recordings and voltages are given as the negative of the patch pipette potential. Negative potentials represent hyperpolarizations while positive potentials represent depolarizations of the cell membrane away from the resting potential. The product of the open probability and the number of functional channels was calculated using pCLAMP 10 software (Molecular Devices) and this represents a measurement of ENaC activity within a patch. For examining ENaC activity in freshly isolated split-open tubules, kidney tubules were dissected from SV129 wild-type mice and the cortical collecting duct was morphology identified. The tubules were split-open in physiological saline in a tissue culture dish before patching the apical surface of the cells.

### Immunoprecipitation

Exosomes were lysed in RIPA buffer supplemented with protease and phosphatase inhibitors. The protein content was measured using the BCA assay (Thermo Scientific; Waltham, MA). Two hundred micrograms of total protein was incubated with a 1:250 dilution of anti-ENaC alpha, beta, or gamma antibody at 4°C for 4 hours with end-over-end rocking. The resulting complexes were incubated with a 1:10 dilution of prewashed 50% slurry of protein A agarose (Thermo Scientific) at 4°C for 8 hours with end-over-end rocking. The resulting complexes were washed four times with ice cold lysis buffer. Bound proteins were eluted by boiling the samples for 10 minutes in Laemmli sample buffer before loading 40 μl of the supernatant on SDS-PAGE gels.

### SDS-PAGE and Western blotting

Cells were lysed in mammalian protein extraction reagent (Thermo Scientific) supplemented with proteases/phosphatase inhibitors and the proteins were resolved on 4–20% Criterion precast gels (BIO-RAD) by SDS-PAGE and then transferred to nitrocellulose membranes as previously described [19]. The membranes were blocked in 5% milk TBS before being incubated with anti-GAPDH antibody (Cell Signaling Tech) at a 1:1000 dilution in 5% BSA TBS and then at a 1:3000 dilution with goat-anti rabbit secondary antibody (BIO-RAD). The membranes were incubated with SuperSignal Dura chemiluminescent substrate (Thermo Scientific) and developed using a BioRad imager.

### Statistical Analysis

Statistical analysis was performed using SigmaPlot (Systat Software, Inc., Point Richmond, CA). Error bars represent standard error of the mean (SEM). We assigned statistical significance at p < 0.05.

## Results

### Characterization of exosome size by electron microscopy and NanoSight analysis

**Fig 1:**
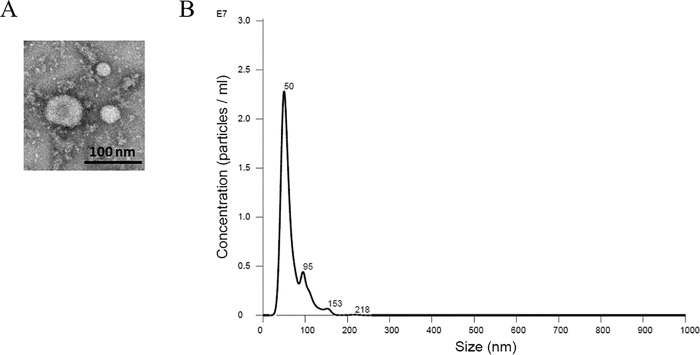
Size characterization of intact exosomes isolated from the apical side of LLC-PK1 cells. (A) Electron micrograph of exosomes purified from LLC-PK1 cells. Negative staining transmission electron microscopy shows a field of exosomes ranging from 30–100 nm in size. Bar, 100nm. (B) Confirmation of exosomes size distribution and concentration by NanoSight analysis. Samples were prepared to a 1:1000 dilution in 1X phosphate buffered saline before being loaded onto a NanoSight NS300 with a high sensitivity camera and green 532nm laser. The size distribution profiles for the purified exosomes showed a peak at 50 nm. The concentration of the exosomes with a diameter of 50 nm in size was 2–2.5x10^7 exosomes/ml.

Microvesicles and exosomes are two forms of extracellular vesicles that are formed by different mechanisms and have distinct characteristics. Microvesicles are plasma-membrane-derived fragments that are shed from various cell types [35]. Conversely, exosomes are formed from multivesicular bodies [35]. Microvesicles range from 200 to 1,500 nm in size [35] while exosomes are generally smaller and range from 30 to 150 nm in size [36]. Negative staining electron microscopy was used to determine the size of a random field containing exosomes isolated from the conditioned media within the apical compartment of permeable transwell inserts used to culture LLC-PK1 proximal tubule cells (Fig 1A). The electron micrograph shows exosomes in the range of 30–100 nm in diameter. In order to corroborate these results and to determine exosome concentration in our samples, we performed NanoSight analysis. The size distribution showed a peak at 50 nm with a concentration of between 2x10^7^ to 2.5x10^7^ exosomes/ml after being diluted 1:1000 (Fig 1B).

### Coomassie Staining and Mass spectrometry analysis of exosomes

**Fig 2:**
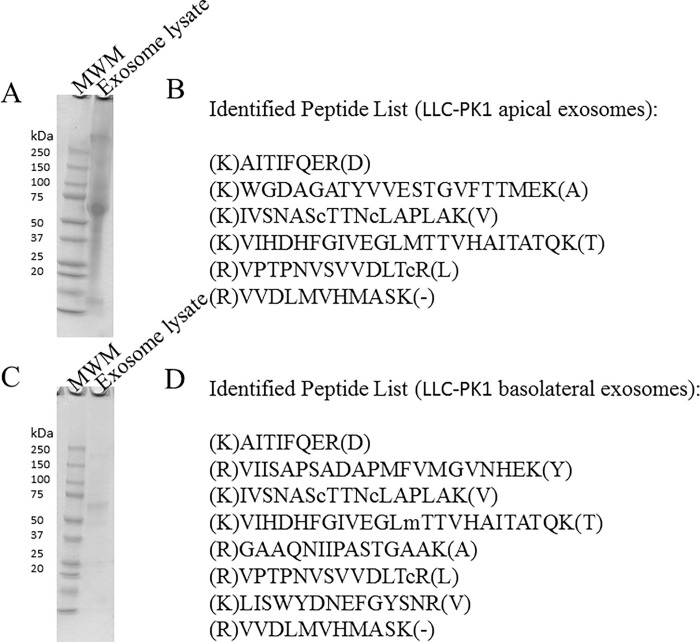
Coomassie blue–stained sodium dodecyl sulphate-polyacrylamide gel electrophoresis analysis (A) and mass spectrometry analysis (B) of lysed exosomes isolated from conditioned media in the apical compartment of LLC-PK1 cells. Coomassie blue–stained sodium dodecyl sulphate-polyacrylamide gel electrophoresis analysis (C) and mass spectrometry analysis (D) of lysed exosomes isolated from conditioned media in the basolateral compartment of LLC-PK1 cells. Molecular weight markers (MWM) are shown in the first lane. Peptides listed in (B) and (D) are signature peptides corresponding to GAPDH.

**Fig 3:**
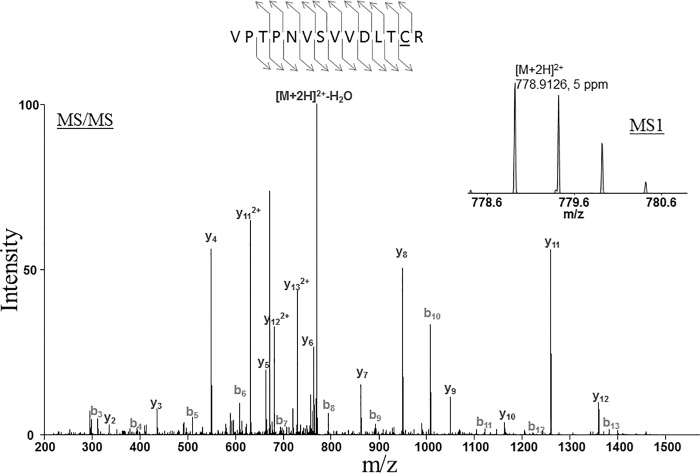
Identification of Glyceraldehyde-3-phosphate dehydrogenase by LC-MS/MS. The peptide was detected as doubly charged with a mass-to-charge ratio of 778.9126, which represents an error of 5 ppm. The tandem mass spectrum matched the following sequence, VPTPNVSVVDLTCR. The detection was made with Mascot with ion score 87.8.

The composition of exosomes isolated from the conditioned media within the apical or basolateral compartments of permeable transwell inserts used to culture LLC-PK1 proximal tubule cells was analyzed by SDS-PAGE and Coomassie staining. The abundance and differential expression of proteins from the exosomal lysates are shown in Fig 2. GAPDH was identified in exosomes isolated from conditioned media within the apical and basolateral compartments of permeable transwell inserts (Fig 2, Fig 3).

### Exosomes from LLC-PK1 cells contain active GAPDH

**Fig 4:**
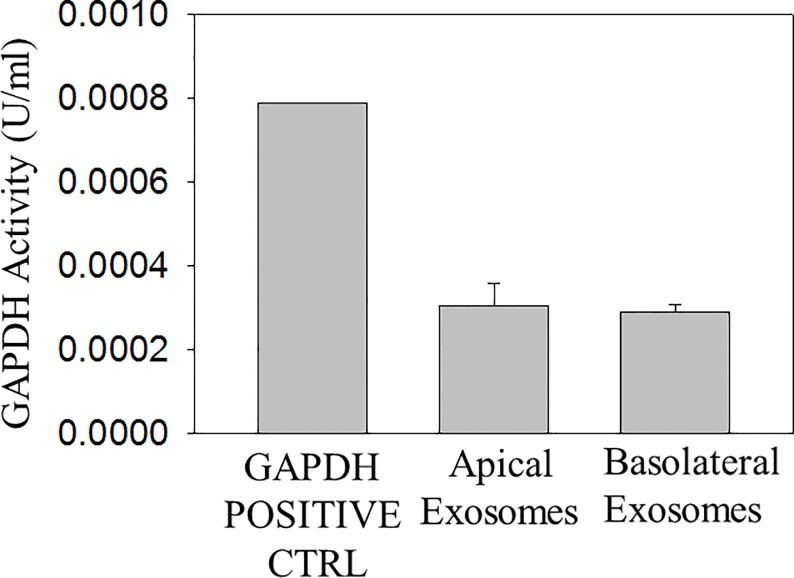
GAPDH activity in exosomes isolated from LLC-PK1 cells. Three batches of exosomes (n = 3) isolated from conditioned media of LLC-PK1 cells present in the apical compartment or basolateral compartment of permeable transwell inserts were lysed in RIPA buffer. The ΔOD was calculated for the GAPDH activity in each sample by taking the difference in absorbance readings between two time points that fell within an NADH standard curve generated using six different concentrations of NADH. All sample readings were corrected for by subtracting the background reading from measuring the OD of the background control mix alone. The NADH amount generated by GAPDH activity during the reaction time was calculated for each sample. GAPDH activity is expressed as U/ml where one unit of GAPDH is the amount of enzyme that will generate 1.0 μmol of NADH per minute at pH 7.2 at 37°C. OD represents optical density. Data is presented as mean ± s.e. from 3 separate batches of exosomes (N = 3).

The exosome lipid bilayer may play an important role in maintaining the integrity of its cargo and protecting the cargo from degradation. The exosome lipid bilayer together with its hydrophilic core may allow GAPDH to remain active inside the exosomes. We lysed exosomes isolated from proximal tubule LLC-PK1 cells by resuspending the pellet in RIPA buffer and then we measured GAPDH activity in vitro. GAPDH catalyzed the conversion of Glyceraldehyde-3-Phosphate to 1, 3-Bisphosphate Glycerate and an intermediate, which reacted with a developer to form a colored product that absorbed maximally at 450 nm. GAPDH activity was detected in exosomes isolated from the apical and basolateral compartments of proximal tubule LLC-PK1 cells (Fig 4).

### Exosomes derived from LLC-PK1 cells are taken up by mpkCCD cells

**Fig 5:**
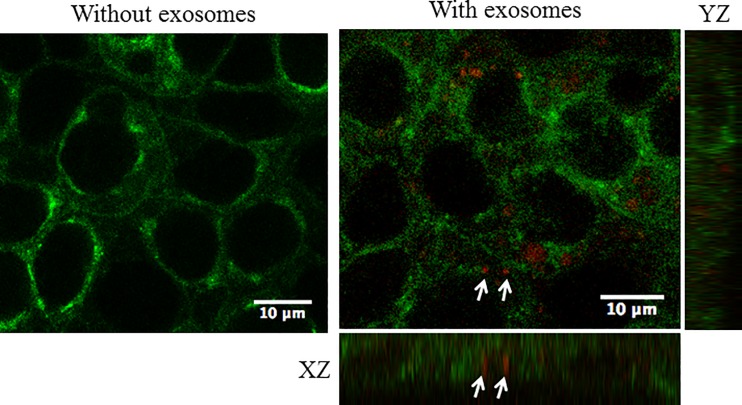
Uptake of fluorescently labeled exosomes from LLC-PK1 cells in mouse cortical collecting duct principal cells. mpkCCD cells were incubated with red PKH26-labeled exosomes at 37°C for one hour. The cell surface was labeled with the green CTX fluorescent dye. Arrows indicate exosomes taken up by the cells after a 1 hour incubation at 37°C. Twelve z stack images were taken in total and an orthogonal view (XZ and YZ axis) is shown.

The ability of exosomes to regulate cellular and signaling pathways depends on the vesicles being taken-up by recipient cells. In order to determine whether distal tubule cells can incorporate exosomes derived from proximal tubule cells we labeled freshly isolated exosomes from proximal tubule LLC-PK1 cells with the red fluorescent dye PKH26. Next, we stained mpkCCD cells with CTX coupled to a green fluorophore in order to label the cell membranes. The exosomes from the donor LLC-PK1 cells were applied to the apical side of permeable transwell inserts containing a monolayer of mpkCCD recipient cells cultured for 10 days to allow for the formation of tight junctions. The cells were imaged by confocal microscopy 1 hour after application of the labeled exosomes. Labeled exosomes are shown to be distributed within the cells after 1 hour (Fig 5).

### Proximal tubule exosomes decrease transepithelial current across mpkCCD cell monolayers

**Fig 6:**
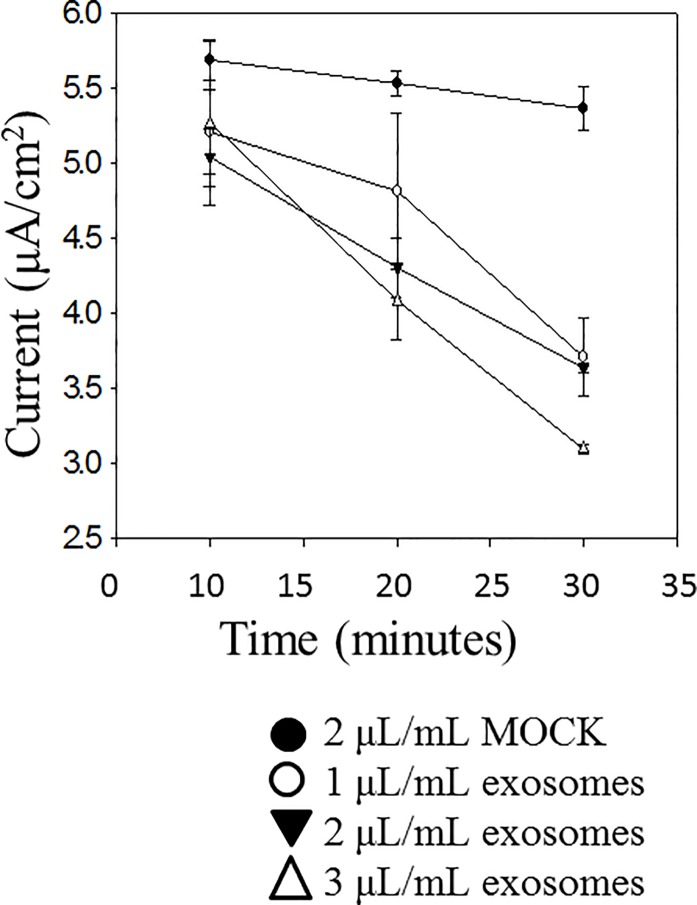
Effect of exogenous application of apical exosomes from LLC-PK1 cells on amiloride-sensitive transepithelial current measurement in mpkCCD cells. Cells were cultured for 10 days to allow the formation of tight junctions and the measurement of resistances and voltages across confluent monolayers. Exosomes isolated from conditioned media present on the apical side of LLC-PK1 cells cultured on permeable transwell inserts were applied to the apical side of mpkCCD cell monolayers. Application of these exosomes resulted in a time and dose dependent decrease in transepithelial current. Each point represents the mean ± s.e. from 3 separate inserts containing cells (N = 3).

**Fig 7:**
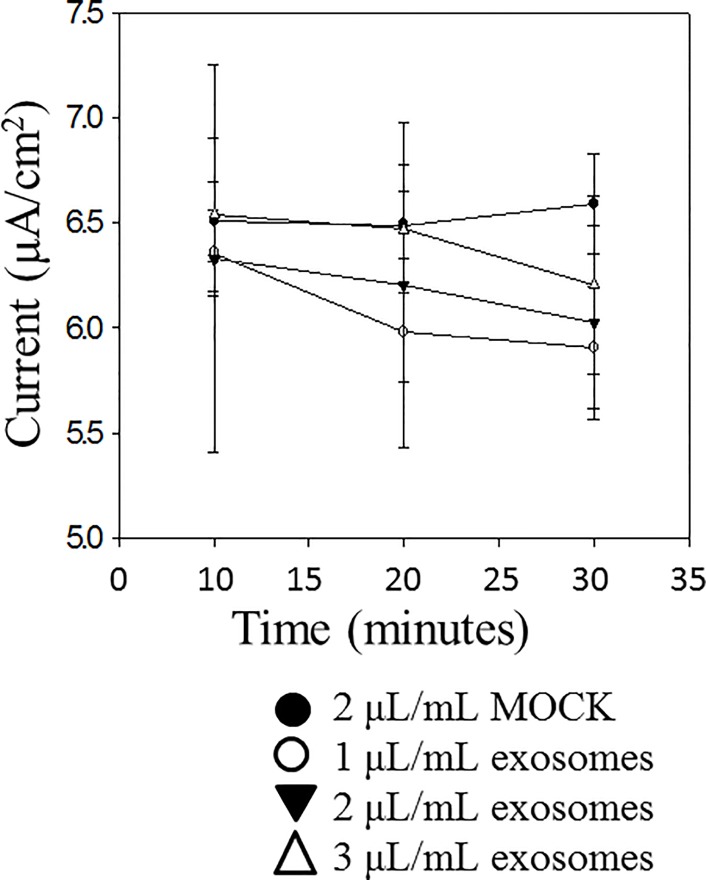
Effect of exogenous application of basolateral exosomes from LLC-PK1 cells on amiloride-sensitive transepithelial current measurement in mpkCCD cells. Cells were cultured for 10 days to allow the formation of tight junctions and the measurement of resistances and voltages across confluent monolayers. Exosomes isolated from conditioned media present on the basolateral side of LLC-PK1 cells cultured on permeable transwell inserts were applied to the apical side of mpkCCD cell monolayers. Application of these exosomes resulted only in a modest decrease in transepithelial current over time. Each point represents the mean ± s.e. from 3 separate inserts containing cells (N = 3).

Cultured mpkCCD cells are capable of generating measurable voltage across a monolayer when grown to confluency and allowed to form tight junctions. Amiloride-sensitive transepithelial current across the monolayers can be calculated using Ohm’s law from the difference in voltage and resistance after application of nanomolar concentrations of amiloride. We examined the effect of exosomes secreted across the apical plasma membrane of LLC-PK1 proximal tubule and exosomes secreted across the basolateral plasma membrane of the same cells on amiloride-sensitive transepithelial current. Exogenous application of exosomes isolated from the apical side of LLC-PK1 cells to the apical surface of mpkCCD cells resulted in a time and dose dependent decrease in amiloride-sensitive transepithelial current (Fig 6) while the effect from exosomes isolated from the basolateral side of LLC-PK1 cells was less pronounced (Fig 7)

### Proximal tubule exosomes decreases ENaC activity in 2F3 distal tubule cells

**Fig 8:**
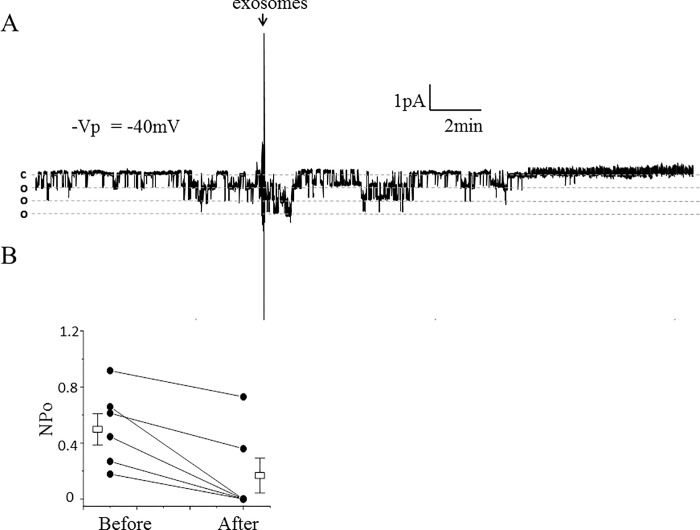
Effect of exosomes isolated from conditioned media on the apical side of LLC-PK1 cells on ENaC activity in Xenopus 2F3 cells. (A): representative single-channel recording using the cell-attached configuration shows a decrease in ENaC activity after application of apical plasma membrane LLC-PK1 exosomes to the apical side of Xenopus 2F3 cells. The dashed lines denote open and closed levels (o, open and c, closed). The number of current levels represents the number of channels in the patch. The arrow indicates the time point at which the exosomes were applied to the cells. (B): summary line graph showing the open probability (*P*_**o**_) of ENaC decreased within 10 minutes of applying exosomes to the apical surface of Xenopus 2F3 cells. Each point represents the mean ± s.e. and the data shown are from 6 separate patches (N = 6); P = 0.07.

**Fig 9:**
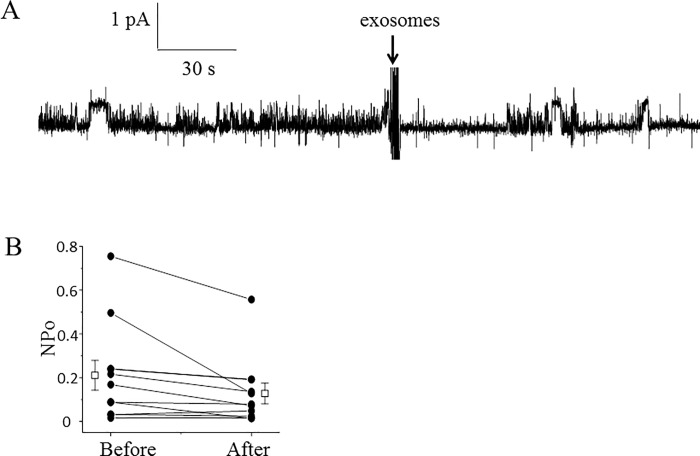
Effect of exosomes isolated from conditioned media on the basolateral side of LLC-PK1 cells on ENaC activity in Xenopus 2F3 cells. (A): representative single-channel recording using the cell-attached configuration shows no appreciable change in ENaC activity after application of basolateral plasma membrane LLC-PK1 exosomes to the apical side of Xenopus 2F3 cells. The dashed lines denote open and closed levels (o, open and c, closed). The number of current levels represents the number of channels in the patch. The arrow indicates the time point at which the exosomes were applied to the cells. (B): summary line graph showing the open probability (*P*_**o**_) of ENaC did not change after applying exosomes to the apical surface of Xenopus 2F3 cells. Each point represents the mean ± s.e. and the data shown are from 10 separate patches (N = 10); P = 0.33.

In order to determine if exosomes derived from proximal tubule cells can alter ENaC activity in cells of the distal tubule we used Xenopus 2F3 cells for single-channel patch clamp studies. Xenopus 2F3 cells were cultured in the presence of aldosterone and endogenously express ENaC alpha, beta, gamma subunits. We isolated exosomes from the conditioned media of the apical or basolateral compartments of permeable transwell inserts used to culture the LLC-PK1 cells proximal tubule cells. We observed a decrease in ENaC activity within 10 minutes of exogenous application of the exosomes to the apical side of Xenopus 2F3 cells (Fig 8). Exosomes derived from the apical side of the LLC-PK1 proximal tubule cells had a greater effect on ENaC activity than the exosomes derived from the basolateral side of the same type of cells (Fig 9).

### Proximal tubule exosomes decreases ENaC open probability in split open tubules

**Fig 10:**
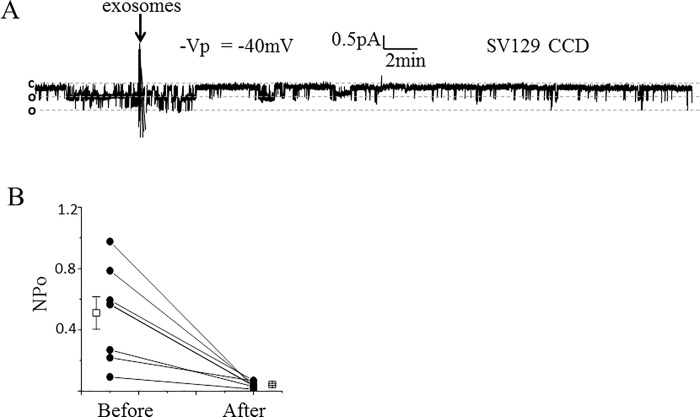
Effect of ENaC activity in freshly isolated split-open tubules from SV129 wild-type mice after application of apical plasma membrane LLC-PK1 exosomes. (A) Representative single-channel recording showing a decrease in ENaC activity in native mouse collecting duct cells after application of exosomes isolated from conditioned media within the apical side of LLC-PK1 cells. The dashed lines denote open and closed levels (o, open and c, closed). The number of current levels represents the number of channels in the patch. The arrow indicates the time point at which the exosomes were applied. (B) summary line graph showing the open probability (*P*_**o**_) of ENaC decreased after applying exosomes isolated from proximal tubule cells. Each point represents the mean ± s.e. and the data shown are from 8 separate patches (N = 8). Two data points are similar and the lines overlap; *P<0.01.

We examined whether ENaC activity in native renal mouse tubules can be regulated by exosomes isolated from proximal tubule cells. Cortical collecting ducts from SV129 wild-type mice were dissected, split open, and principal cells were subject to cell-attached patch clamp studies. Single-channel analysis in freshly isolated split-open tubules showed a decrease in ENaC activity at the level of its open probability compared to basal ENaC activity after application of exosomes isolated from proximal tubule cells (Fig 10).

### Inhibition of GAPDH blunts the exosome-mediated decrease in ENaC activity

**Fig 11:**
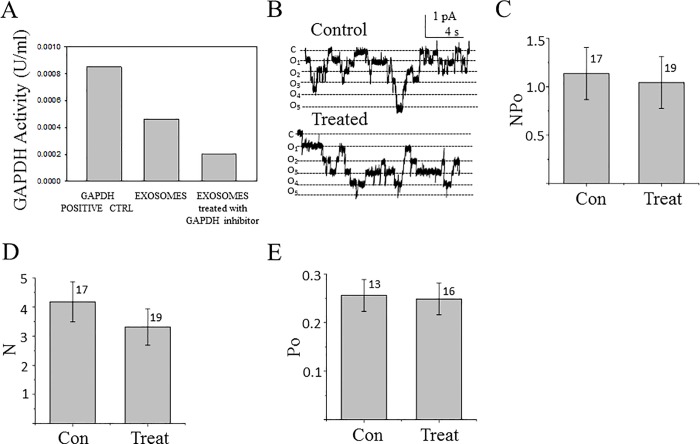
Effect of apical plasma membrane LLC-PK1 exosomes with GAPDH depleted activity on ENaC activity in Xenopus 2F3 cells. (A) GAPDH activity was measured after lysing apical plasma membrane LLC-PK1 exosomes treated with the potent and selective GAPDH inhibitor Heptelidic acid (Koningic acid). (B) Representative single-channel patch clamp recording from the control and treated group. (C-E) Summary of all single channel data. The summary bar graph in (C) shows ENaC activity, NPo. The summary bar graph in (D) shows the number of channels, N. The summary bar graph in (E) shows the open probability, Po for the control and treated groups. Data is presented as the mean ± s.e. and the number of patches is indicated above each group.

Next, we confirmed GAPDH is a molecule within exosomes that can negatively regulate ENaC activity. We transfected proximal tubule apical plasma membrane derived exosomes with the selective and potent inhibitor of GAPDH, heptelidic acid (koningic acid) before applying these modified exosomes to Xenopus 2F3 distal tubule cells and patching for ENaC activity. As shown in Fig 11A, transfection of exosomes with heptelidic acid resulted in a decrease in GAPDH activity. When the effect of these GAPDH depleted exosomes was examined by single-channel patch clamp studies there was no change in ENaC activity between the control and exosome treated groups (Fig 11B–11E).

### GAPDH associates with ENaC subunits

**Fig 12:**
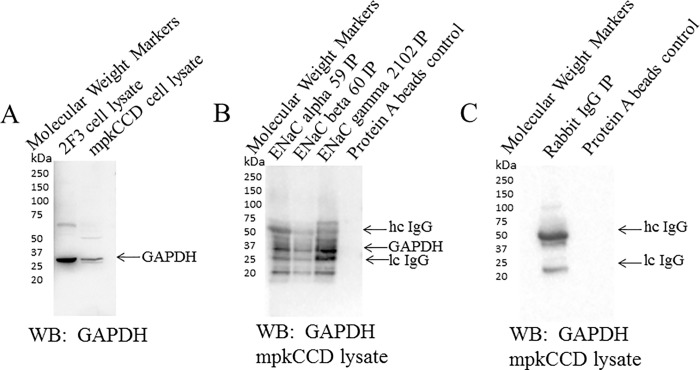
Coimmunoprecipation and Western blot analysis showing an association between GAPDH and ENaC subunits. Polyclonal ENaC alpha, beta, and gamma antibodies were used to immunoprecipatate each subunit from mpkCCD cellular lysates and pull-down protein binding partners. The eluent from the immunoprecipitated complexes were separated by SDS-PAGE and the blots were probed with GAPDH polyclonal antibody. (A) Western blot analysis showing an immunoreactive band corresponding to GAPDH protein at 35 kDa from both Xenopus 2F3 cells and mpkCCD cells. (B) IP-Western showing specific antibodies for ENaC alpha, beta, and gamma subunits pull-down GAPDH from mpkCCD cell lysates. Heavy and light chains of IgG are indicated by arrows. IP refers to immunoprecipitation. IgG refers to immunoglobulin.

ENaC alpha, beta, and gamma subunits have a similar topology consisting of a large extracellular loop flanked by hydrophobic transmembrane domains, and a short amino terminus at one end and a short carboxy terminus at the other end. It is possible for either or both of the cytoplasmic amino and carboxy termini of ENaC subunits to bind endogenous intracellular GAPDH protein. In order to determine whether GAPDH associates with ENaC to allow for its regulation, we used mpkCCD lysate as a source of endogenous ENaC and GAPDH proteins to examine a possible association between the two proteins. Polyclonal antibodies specific for each ENaC subunit were used to immunoprecipitate ENaC and pull-down associated proteins. These antibodies were previously characterized [28] and it was demonstrated that they can be used to enrich for each specific ENaC subunit [23]. The blots were then probed for GAPDH to identify an association between ENaC subunits and GAPDH. ENaC alpha, beta, and gamma subunits were found to associate with GAPDH in mpkCCD cells (Fig 12).

## Discussion

Here we demonstrate a potential role for exosomes in the kidney. We show exosomes from donor proximal tubule cells can regulate ENaC activity in recipient distal tubule and collecting duct cells. We show active GAPDH is present in exosomes isolated from LLC-PK1 proximal tubule cells and we discuss possible mechanisms by which GAPDH can regulate the open probability of ENaC after being taken up by cells of the distal tubule and collecting duct.

There are multiple possible mechanisms that could explain the ability of GAPDH to decrease ENaC activity by reducing the channel’s open probability. First, an increase in GAPDH activity might stimulate an increase in intracellular calcium. Patterson et al showed activation of GAPDH increases NADH levels leading to IP3R mediated calcium release [31]. We and others have shown increases in intracellular calcium can modulate ENaC activity at multiple levels. The MARCKS mediated PIP2 dependent regulation of ENaC is sensitive to changes in calcium and calmodulin. We have previously shown increases in intracellular calcium results in calcium-calmodulin translocation to the apical plasma membrane and subsequent MARCKS displacement from the membrane in Xenopus 2F3 cells [19]. The ability of MARCKS to sequester and present PIP2 for ENaC regulation is dependent on its expression at the apical membrane. We also showed the association between filamin and ENaC subunits is attenuated by increases in intracellular calcium [19]. We showed the phosphorylation of filamin by CaMKII causes a reorganization of the actin cytoskeleton and decrease in ENaC activity [19]. Secondly, an increase in GAPDH activity may increase the local concentration of NADH and adenosine triphosphate (ATP) [31]. Ma et al showed exogenous ATP can inhibit ENaC activity via a P_2_ receptor coupled to a PLC-mediated pathway [37]. Finally, GAPDH may cause a confirmational change in ENaC due to direct binding between the two proteins.

Immunoprecipitation studies presented here show an association between GAPDH and ENaC subunits. ENaC functions most efficiently as a heterotrimeric complex of alpha, beta, and gamma subunits. The interaction between GAPDH and ENaC may cause the ENaC subunits to disassociate or associate with proteins with an inhibitory effect.

It is not surprising GAPDH interacts with cytoskeletal elements within the cell. GAPDH is a cytoplasmic protein that is ubiquitously expressed across different cell types. Intriguingly, its function is not limited to glycolysis but instead GAPDH has been shown to participate in the regulation of cytoskeleton dynamics [38], apoptosis [39], endocytosis [40], membrane fusion [41], and vesicular transport [42]. The multiple roles of GAPDH are regulated by oligomerization, subcellular localization, and various posttranslational modifications [43].

This is the first report of the amiloride-sensitive renal epithelial sodium channel being regulated by exosome derived GAPDH. Renigunta et al previously showed the renal epithelial potassium channel (ROMK2) is regulated by GAPDH and enolase [44]. This indicates transport mechanisms in the kidney are sensitive to glycolytic enzymes. The steady-state level of glycolytic enzymes such as GAPDH is kept relatively constant within the cell. Presumably, exosomal delivery of GAPDH into the cell increases the intracellular concentration of active GAPDH near the inner leaflet of the apical plasma membrane to decrease ENaC activity.

Although we were able to show collecting duct principal cells can take-up exosomes originating from proximal tubule cells, we have not identified the exact mechanism for the incorporation of the exosomes into the cells or demonstrated specificity of the exosomes to target a particular cell type. It is possible the exosomes express surface peptides that are involved in cell recognition and incorporation into the recipient cell.

The proximal tubule is the site where the majority of water and ions are reabsorbed back into the body. The physiological significance of exosomes derived from proximal tubule cells regulating ENaC activity is underscored by the role of this channel in the fine tuning of sodium in the distal tubule and collecting duct. The present study suggests exosomes may play an important role in communication within the nephron.
